# Epidemiological profile of extremity fractures in victims of motorcycle accidents

**DOI:** 10.1590/1413-78522015230100998

**Published:** 2015

**Authors:** Flamarion dos Santos Batista, Leandro Oliveira Silveira, Jesús José André Quintana Castillo, Jady Elen de Pontes, Luz Delícia Castillo Villalobos

**Affiliations:** I Hospital Universitário Evangélico de Curitiba, Curitiba, PR, Brazil, Hospital Universitário Evangélico de Curitiba, Curitiba, PR, Brazil; II Faculdade Evangélica do Paraná, Curitiba, PR, Brazil, Faculdade Evangélica do Paraná, Curitiba, PR, Brazil; III Universidade Tecnológica Federal do Paraná, Curitiba, PR, Brazil, Universidade Tecnológica Federal do Paraná, Curitiba, PR, Brazil

**Keywords:** Fractures, bone, Accidents, traffic, Motorcycles

## Abstract

**Objective:**

Show the epidemiological profile of limb fractures in victim of motorcycle accident seen at the Emergency Department of Hospital Universitário Evangélico de Curitiba (HUEC), Curitiba, PR, Brazil, from January 2007 to December 2013, as well as to compare the results with data from the literature.

**Methods:**

This is a retrospective, descriptive, observational study. The information was obtained from the analysis of all the medical records from January 2007 to December 2013 belonging to the hospital archives. Only extremity fractures cases from motorcycle accident victims were analyzed, according to the medical records and radiological reports. The ICD-10 was used as classification criterion, and the fractures were grouped depending on the topography of the injury. The following variables were considered: number of victims, gender, age and fracture site, in order to create a database to contrast with the literature.

**Results:**

During seven years, 3,528 motorcycle accident victims have been identified, 88.29% being male, whereas 11.71% being female. The average age of the victims was 29.7 years old, observing a strong inverse correlation between the number of victims and their ages. There has been 4,365 fractures, being 59.66% in lower limbs and 40.34% in upper limbs. From that total, 18.14% were leg fractures, 11.57% were hand fractures and 10.65% were wrist fractures.

**Conclusion:**

This study has met its objectives and the results were similar to the national literature. Level of Evidence II, Retropective Study.

## INTRODUCTION

Motorcycles are an easy to purchase means of transportation as compared to cars. Cheaper and more economical, their number grows exponentially in the streets and highways of Brazil, reaching 16.9 million in circulation in the year 2012.^1^

The finding that the traffic of Curitiba, PR, Brazil, which has been known as "planning model", is in chaos, due to the rapid increase of the vehicle fleet, is a concern regarding accidents involving motorcyclists. Due to their large exposure to risks, the high demand for businesses goods and services and often due to disobedience to traffic laws, motorcycle accidents have more serious consequences. Cabral *et al.* 2 observed that, among the 1,032 individuals who suffered accident due to ground transportation, in 57.4% of cases motorcycles were involved in the accident scene.

In a study conducted in an emergency care service with 90 motorcyclists victims of traffic accidents in Porto Alegre, RS, Brazil most injuries were fractures of limbs or other injuries leading to temporary incapacity of the victims.^3^ Therefore, to establish the number of each type of fracture resulting from these accidents which presents to public emergency service is extremely important for the proper preparation of these services.

In the same study, it has been found that fractures of the lower limbs were the most frequent type of injury in those victims (22.60% of all injuries), followed by bruises (19.56%), fractures of the upper limbs (8.90%), chest trauma (6.85%) and traumatic brain injury (6.17%).3 In a study conducted in 2003 in the city of Maringá, PR, Brazil, with a sample of 67 victims, it was found that most of the injured were men (86.57%), in most productive group, aged between 14 and 32 years old (71.64%), and mostly with injuries on lower limbs, followed by injuries in upper limbs.^4^ In 2000, in Uberlândia, MG, with 965 victims, limb fractures totaled 31.84% of the injuries, and 143 injured had at least one fracture, while 28 had multiple fractures.^5^

The objective of this study is to show the epidemiological profile of limb fractures in accidents by motorcycle treated at the *Hospital Universitário Evangélico de Curitiba* (HUEC), Curitiba, PR, Brazil, from January 2007 to December 2013 and compare the results with data from the literature.

## METHODS

This is an observational, descriptive and retrospective study. Data were obtained through the analysis of all records, from January 2007 to December 2013, at the Orthopedics and Traumatology Service, cataloged at the Medical File of *Hospital Universitário Evangélico de Curitiba*.

In the present study only limb fractures of victims of motorcycle accidents, according to medical records and radiology reports, were analyzed.

As a classification criterion we analyzed the presented radiographies, observing the fracture trace regarding the anatomical region, according to the International Classification of Diseases (ICD-10). The fractures were grouped according to the topography of the injury: shoulder fractures; arm fractures; elbow fractures; forearm fractures; wrist fractures; hand fractures; pelvic fractures; hip fractures; thigh fractures; knee fractures; leg fractures; ankle fractures; and foot fractures.

The following variables were analyzed: number of victims, gender, age and type of fractures. Incomplete, illegible or containing conflicting information records were excluded. In addition, only victims between 18 and 60 years old have been selected. Data was, then, compared to the related literature.

A database was made for registration of information, consisting of the variables year, age, gender, type of fractures and a patient identification number, just for organization purposes. The software used for this purpose was Microsoft Excel 2007, and the graphics and statistical calculations were made using this software.

## RESULTS

Out of a total of 3,528 patients' records from the Orthopedics and Traumatology ward of *Hospital Universitário Evangélico de Curitiba*, treated between January 2007 and December 2013 were analyzed. The total number of fractures encountered, according to ICD-10, was 4,365 fractures, since some victims had more than one fracture. (Table 1)

**Table 1.** Number of victims and number of limb fractures on the period of study, per year.

Year	N of victims	N of fractures
2007	389	479
2008	544	698
2009	418	518
2010	499	609
2011	584	707
2012	613	781
2013	481	573
Total	3528	4365

Of the total 3,528 victims studied, 3,114 (88.29%) were males and 414 (11.71%) were females. (Table 2)

**Table 2.** Gender of the victims on the period of study, per year.

Year	Masculine	Feminine
2007	353 (90.75%)	36 (9.25%)
2008	482 (88.60%)	62 (11.40%)
2009	369 (88.28%)	49 (11.72%)
2010	447 (89.58%)	52 (10.42%)
2011	512 (87.67%)	72 (12.33%)
2012	531 (86.62%)	82 (13.88%)
2013	420 (87.32%)	61 (12.68%)
Total	3114 (88.29%)	414 (11.71%)

Regarding the age of the victims, most were between 18 and 28 years old. The mean age was 29.7 years old and the median was 28 years old. The largest number of victims who have suffered some kind of limb fractures was 20 years old. (Table 3)

**Table 3.** Number of victims who suffered some type of limb fractures, per age.

Age (years)	N of victims	Age (years)	N of victims	Age (years)	N of victims	Age (years)	N of victims
18	154	29	143	40	68	51	20
19	202	30	124	41	69	52	22
20	214	31	102	42	57	53	15
21	199	32	101	43	51	54	10
22	206	33	81	44	53	55	13
23	171	34	85	45	38	56	11
24	175	35	76	46	40	57	5
25	178	36	93	47	32	58	7
26	140	37	70	48	37	59	7
27	123	38	65	49	28	60	7
28	151	39	62	50	23	Total	3528

**Figure 1.**
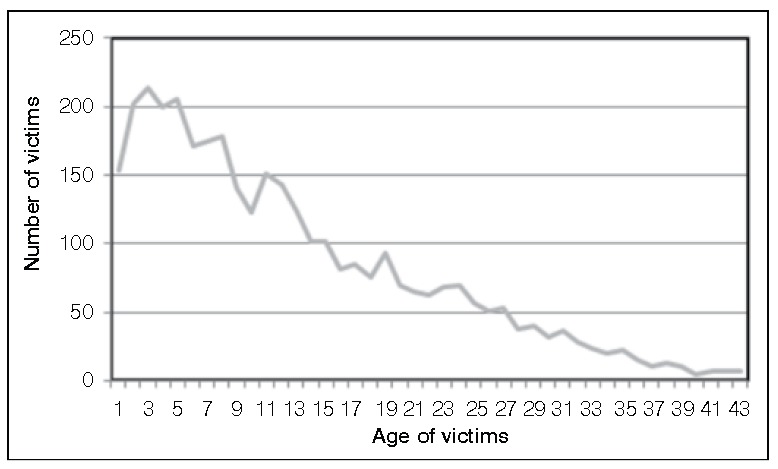
Number of victims who suffered some type of fractures according to age.

In this situation, it has been observed that the victims age in relation to the number of victims is strongly and inversely correlated, with r = -0.9587. (Figure 1)

The evaluation of prevalence of limb fractures was performed according to the work methodology. We found 2,604 lower limb fractures (59.66%) and 1,761 upper limb fractures (40.34%). (Table 4)

**Table 4.** Frequency of fractures in upper and lower limbs.

Upper limbs		Lower limbs	
Shoulder	460 (10.54%)	Pelvis	121 (2.77%)
Arm	72 (1.65%)	Hip	98 (2.25%)
Elbow	108 (2.47%)	Thigh	449 (10.29%)
Forearm	151 (3.46%)	Knee	361 (8.27%)
Wrist	465 (10.65%)	Leg	792 (18.14%)
Hand	505 (11.57%)	Ankle	425 (9.74%)
-	-	Foot	358 (8.20%)
Total Upper limbs	1761 (40.34%)	Total Lower limbs	2604 (59.66%)
Grand Total		4365 (100%)	

**Figure 2.**
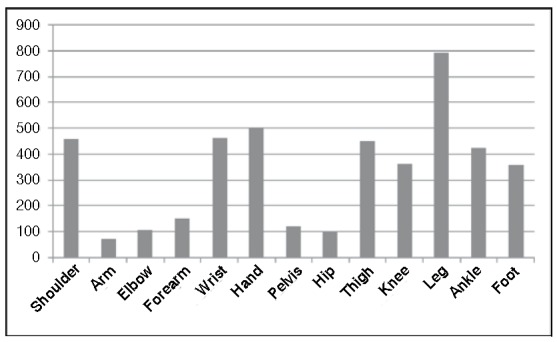
Frequency of fractures regarding the topographic region.

The most prevalent type of fractures was the leg fracture, with 792 cases or 18.14% of the overall total. Then, with 505 fractures, comes hand fractures, totaling 11.57%. The third most prevalent type was wrist fractures, 10.65% (465 fractures), followed by shoulder fractures with 460 and thigh fractures with 449. Following the order of prevalence, then come ankle fractures, knee, foot, forearm, pelvis, elbow, hip and arm fractures. (Figure 2)

**Figure 3.**
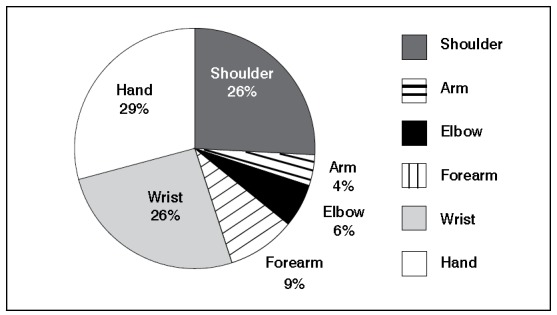
Prevalence of fractures by topographic region (only upper limbs).

**Figure 4.**
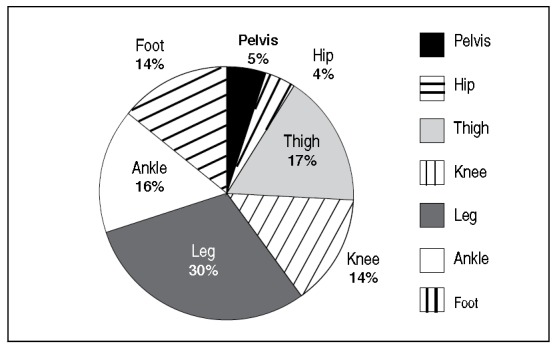
Prevalence of fractures by topographic region (only lower limbs).

In Figures 3 and 4 we observe the fracture prevalence comparing isolated only upper and only lower limbs, respectively.

## DISCUSSION

The prevalence of adult males observed in this work agrees to what has been reported in several similar studies. In our study, we found that 88.86% of the victims were men. In the study by Pinto and Witt,^3^ this representation was 86.7%, in Dall'aglio^5^ it was 77.78%, in Santos *et al.* 6 it was 87.43%, and in Silva *et al.* 7 it was 83, 5% of all casualties. The prevalence of motorcycle accidents involving men may be related to their greater exposure, as well as greater use of such vehicle.^7^

Regarding age, the most prevalent was 20 years old, representing 6.06% of the victims. The mean age was 29.7 years old and the median 28 years old. Dall'aglio5 has demonstrated in his study that 63.89% of the accident victims were between 15 and 40 years. However, Pinto and Witt3 showed that 78.9% of the victims were in the range between 18 and 35 years old.

With the foregoing statements in these studies, there was a close relationship of economically active population and potentially contributing labor class with motorcycle accidents. The fact that the prevalence of the victims focuses on young male adult favors an exclamation point in the economic situation of the country. From this representation, it implied a very high cost on the recovery of these patients and a consequent fall in the contribution to the Union due to their withdrawal from work.

For the motorcyclists, limbs are precisely the most vulnerable regions, since the safety equipment provides protection only to head region.^4^ The injuries resulting from traffic accidents vary greatly depending on the trauma kinematics in each situation. In the case of motorcycles, usually collisions are frontal, lateral or from rear. Falls also play an important role in the occurrence of injuries.

The total number of fractures was found in 4,365 in a seven years period. Pinto and Witt^3^ found 54 cases from March to April 2007. It is important to notice that, in both studies, the chosen hospital was a Reference Centre for Trauma Care in a State Capital.

Regarding upper limb fractures (shoulder, arm, elbow, forearm, wrist and hand), 1,761 fractures were identified, corresponding to 40.34% of all fractures. Regarding lower limb fractures (pelvis, hip, thigh, knee, leg, ankle and foot), we identified 2,604 fractures, corresponding to 59.66%. Pinto and Witt3 observed the prevalence of 38.89% and 61.11%, respectively, for upper and lower limb fractures.

Brazil, becoming increasingly chaotic in urban mobility, needs to adjust to enable more quality and safety for motorcyclists and transit users in general. The epidemiology, particularly in situations involving the need for better hospital infrastructure, is essential in understanding the demand of patients injured by accidents. Emergency room services should be aware of the most prevalent situations to provide a proper service to traffic victims. If, on the one hand, efforts to minimize the consequences of the accident must be continuously improved, those aimed at the prevention and control of accidents, such as the practice of social welfare, should be prioritized.^8^

Unfortunately, studies with an epidemiological survey of the same extent as this study are rare in the national literature. Adding to this, comparing our results with other ones was very difficult. A serial follow up of the characteristics presented in is highly recommended. Moreover, we suggest to pursue a correlation between fractures found and the trauma mechanism (motorcycle x automobile, motorcycle x motorcycle; motorcycle x shield, among others), in addition to assess the concomitant injuries in victims of such accidents.

## CONCLUSION

From the analysis of the data presented above, we conclude that our study was similar to the national literature consulted. The most prevalent anatomical region in cases of extremity fractures in motorcycle accident victims was the leg. Subsequent prevalence is of upper limb injuries, however, lower limbs as a whole showed more fracture cases. The most observed victim pattern was young male adults.
